# Expansion of Sphingosine Kinase and Sphingosine-1-Phosphate Receptor Function in Normal and Cancer Cells: From Membrane Restructuring to Mediation of Estrogen Signaling and Stem Cell Programming

**DOI:** 10.3390/ijms19020420

**Published:** 2018-01-31

**Authors:** Olga A. Sukocheva

**Affiliations:** College of Nursing and Health Sciences, Flinders University of South Australia, Bedford Park, SA 5042, Australia; olga.sukocheva@flinders.edu.au

**Keywords:** sphingolipids, sphingosine-1-phosphate, sphingosine kinase, estrogen receptor, breast cancer, inflammation, vasculature, cancer stem cells

## Abstract

Sphingolipids, sphingolipid metabolizing enzymes, and their receptors network are being recognized as part of the signaling mechanisms, which govern breast cancer cell growth, migration, and survival during chemotherapy treatment. Approximately 70% of breast cancers are estrogen receptor (ER) positive and, thus, rely on estrogen signaling. Estrogen activates an intracellular network composed of many cytoplasmic and nuclear mediators. Some estrogen effects can be mediated by sphingolipids. Estrogen activates sphingosine kinase 1 (SphK1) and amplifies the intracellular concentration of sphingosine-1-phosphate (S1P) in breast cancer cells during stimulation of proliferation and survival. Specifically, Estrogen activates S1P receptors (S1PR) and induces growth factor receptor transactivation. SphK, S1P, and S1PR expression are causally associated with endocrine resistance and progression to advanced tumor stages in ER-positive breast cancers *in vivo*. Recently, the network of SphK/S1PR was shown to promote the development of ER-negative cancers and breast cancer stem cells, as well as stimulating angiogenesis. Novel findings confirm and broaden our knowledge about the cross-talk between sphingolipids and estrogen network in normal and malignant cells. Current S1PRs therapeutic inhibition was indicated as a promising chemotherapy approach in non-responsive and advanced malignancies. Considering that sphingolipid signaling has a prominent role in terminally differentiated cells, the impact should be considered when designing specific SphK/S1PR inhibitors. This study analyzes the dynamic of the transformation of sphingolipid axis during a transition from normal to pathological condition on the level of the whole organism. The sphingolipid-based mediation and facilitation of global effects of estrogen were critically accented as a bridging mechanism that should be explored in cancer prevention.

## 1. Introduction

Steroid hormone Estrogen is the key physiological factor for normal development and differentiation of mammary and other reproductive tissues [1,2,3]. Nevertheless, in mammary tumors, the estrogen signaling pathway is transformed to facilitate pathological proliferation [1,4]. Considering that mammary cancers are the second leading cause of cancer death in women, all cancer-linked estrogen effects deserve to be thoroughly investigated.

The role of Sphingosine kinase (SphK) as mediator of estrogen-induced growth-promoting effects was discovered in MCF-7 human breast cancer cells more than a decade ago [5,6]. SphK is the enzyme that catalyzes the transformation of sphingosine in sphingosine-1-phosphate (S1P). Two isoforms of SphK, SphK1 and SphK2, were detected and revealed to mediate several distinct biological processes induced by a variety of growth factors, cytokines, and mitogenic factors [7,8]. It was found that increased SphK1 activity coincides with boosted cell growth and serves as a required mediator of estrogen-dependent activation of mitogen-activated protein kinase (MAPK) and intracellular Ca^2+^ mobilization [5]. Apparently, estrogen not only regulates sphingolipid composition and signaling in cancer cells, but also intensifies SphK1 expression. Similarly, the estrogen receptor modulator tamoxifen stimulated expression of SphK1 in antiestrogen resistant breast cancer cells *in vitro* [9]. Increased SphK1 levels were detected in advanced and metastatic breast tumors *in vivo* [10]. It was further suggested that SphK1 can contribute to the development of anti-cancer drug resistance [9,11,12]. Consequently, sphingolipids are considered as novel estrogen and ER-activated cytoplasmic second messengers. However, the details of molecular mechanisms that link estrogen receptors (ERs) and the SphK/S1P signaling network remain to be discovered.

SphK/S1P signaling was shown to interact with a complex growth factor network that facilitates cancer cell proliferation. Estrogen signaling also overlaps with a growth factor receptor network in breast cancer cells [13]. These cross-talk interactions are mutual. Epidermal growth factor receptors (EGFR) not only influence the estrogen pathway and regulate breast cancer cell survival and spreading [13], but also influence the SphK1 network [14,15,16]. Thus, the crosstalk process includes all three components (growth factors, estrogen, and sphingolipid networks) and can be called tripartite. Supporting this, transduction of signals from ER to EGFR were mediated by a third party: the S1PR3 [14]. Activated S1PR3 in turn delayed EGFR degradation in favor of endosomal rotation and EGFR recycling that prolongs positive proliferation stimuli [17].

Thus, sphingolipids play an important mediatory role in estrogen signaling and may account, at least partially, for the uncontrollable growth and division of breast cancer cells. Recently, it was demonstrated that the SphK/S1P axis is involved in breast cancer stem cell functioning [18,19], angiogenesis [20,21], and lymphangiogenesis [22,23]. This review will account for discovered milestones of the SphK/S1P/S1PR signaling axis including the sphingolipids role in maintenance of homeostasis and estrogen-linked responses in normal and malignant cells. It will also discuss the potential directions of future experimental work that should uncover clinically valuable details of sphingolipid signaling crosstalk with major regulatory agents such as hormones, cytokines, and growth factors.

## 2. Sphingosine Kinases, Sphingosine-1-Phosphate, and Membrane Metabolism

Extra- and intra-cellular membranes are dynamic structures that are being constantly renewed to maintain appropriate functionality. The composition of cell membranes is regulated to correspond with specific protein performance and effectiveness [24]. The cell membranes can be extended to give room for growth, or demolished following the turnover process. Specifically, consecutive enzymatic degradation of membrane glycosphingolipids and sphingomyelin generates ceramide and then sphingosine, the precursor substrate for the synthesis of S1P [25,26]. The main yield of sphingosine, a lipid with strong pro-apoptotic properties, is accumulated in lysosomes [27]. To avoid cell death initiation, generated sphingosine should be quickly phosphorylated by sphingosine kinases, SphK1, and/or SphK2, to produce S1P, a pro-survival signaling molecule [8,25,26].

To date, three unique isoforms of the human SphK1 have been identified, differing at the N-terminus: hSphK1a, hSphK1b, and hSphK1c [28]. Two transcriptional SphK1 isoforms, 43 and 51 kDa, have been identified [29]. Most studies do not specify the targeted isoform, although the shorter hSphK1a was conventionally employed and will be referred to as SphK1 in this review. The SphK2 encodes at least four predicted variants (a–d) [30] that remain poorly investigated to date and will be referred to as SphK2.

According to the previous findings, SphK1 is localized in the cytoplasm and can be translocated to the plasma membranes [31], phagosomes [32], endosomes [33], and nucleus [34,35,36]. However, the purpose of SphK1 nuclear presence, although detected by several groups [34,35,36], is not well understood. Yagoub et al. [29] identified common and isoform-specific SphK1-interacting partners including supervillin, drebrin, and the myristoylated alanine-rich C-kinase substrate-related protein that were shown to support cell migration, adhesion, and cytoskeletal remodeling [29]. The SphK1 51 kDa isoform was exclusively localized to breast cancer cells and associated with proteins such as allograft inflammatory factor 1-like protein, the latent-transforming growth factor β-binding protein, and dipeptidyl peptidase 2 [29]. The signaling and/or regulatory roles of these interactions in cancer cell growth and metabolism remain to be uncovered.

SphK2 was more commonly found in the nucleus, although the enzyme has been detected in the cytoplasm also [30,37,38]. SphK2 may translocate to cytoplasm to compensate for SphK1 functions. The isoenzymes’ housekeeping functions to maintain membrane sphingolipid turnover by phosphorylation of sphingosine are considered redundant and often overlap, but separate isoform-specific functions were also demonstrated [39,40], and might be associated with the difference in SphK1/2 intracellular localization. Considering that the enzyme localization often reflects its function, the presence of SphK1 in a different compartment from SphK2 indicates prospective divergence in the isoenzyme specialization and functioning.

Newly synthesized S1P is a multifunctional molecule that can be utilized in several different pathways. Endoplasmic reticulum located phosphatases can dephosphorylate nearly half of the available S1P and direct it back for ceramide synthesis, thus recycling the lipid [27,41,42]. Alternatively, S1P is irreversibly reduced by S1P lyase into phosphoethanolamine and hexadecenal [43], which are further utilized in the glycerolipid pathway. S1P-originated hexadecenal is converted by fatty aldehyde dehydrogenase to hexadecanoate, an originator of palmitoyl-CoA [44]. S1P-derived phosphoethanolamine is also used further for phosphatidylethanolamine synthesis [45,46]. In addition to its structural role, the signaling role of membrane lipid composition has been long recognized [24,47]. However, the questions about regulatory impact and specific signaling targets for the large, diverse group of S1P metabolites have only been recently approached [48], although they remain largely unexplored.

**Figure 1:**
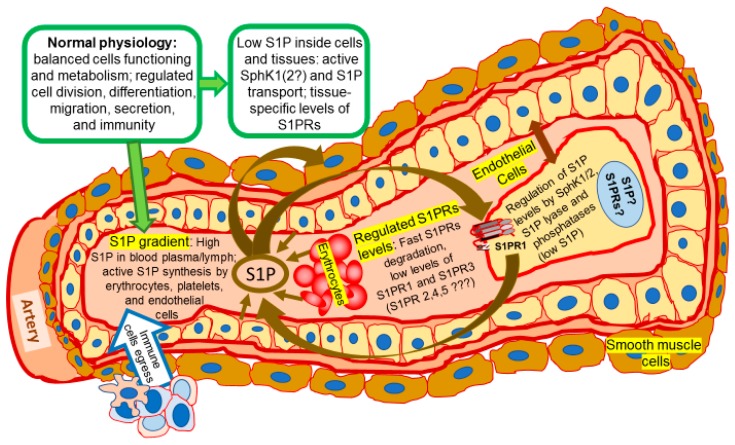
Normal physiology: the place of sphingolipid signaling pathway. A transverse cut through the artery shows an inside space of the blood vessel filled with blood plasma and blood cells (only erythrocytes are shown), as well as a layer of endothelial cells surrounded by smooth muscle cells. Endothelial cell layer and enlarged single endothelial cell are indicated by a double-ended arrow. Coordinated sphingolipid signaling creates a favorable environment for normal physiological functioning. The activity and expression levels of sphingosine kinases (SphK), sphingosine-1-phosphate (S1P) and its receptors (S1PRs) are maintained at normally low tissue specific levels required for healthy metabolism. Alternatively, high concentration of S1P in blood plasma (and lymph) is supported by release of S1P from endothelial cells, erythrocytes, and platelets. Binding of S1P to S1PRs stimulates activation of an appropriate signaling in cytoplasm and gene activation in nucleus that are followed by quick S1PRs internalization, degradation, and re-cycling. Activation of endogenous SphK by hormones, cytokines, and growth factors results in reduction of sphingosine cell content and production of S1P. S1P/S1PRs axis activates further downstream signaling targets and controls variety of physiological processes including lymphocyte egress from lymphoid organs [5,8,9,14,17,21,62,63,64,65,66,67,68]. Arrows indicate the movement of S1P and direction of its effects (such as activation of S1PR by S1P as ligand, release of S1P by endothelial or cancer cells, etc.). The arrow directed from S1P to the endothelial cell indicates that S1P also activates endothelial cell signaling via S1PRs. Question marks indicate the gaps in our knowledge of SphK/S1P/S1PRs axis.

High levels of S1P at sub-micromolar concentrations were detected in circulation [49,50], mainly in complex with high-density lipo-protein (HDL) particles [51]. S1P can be released to the extracellular liquids in quite large quantities by some cells including erythrocytes [52,53,54,55], platelets [56,57], tumor cells [14,58], and endothelial cells [21,53,54,55] (Figure 1). It was found that endothelial cells secrete S1P through a specific S1P transporter, spinster homolog 2 (Spns2) [59,60]. Activated by thrombotic agonists, platelets release S1P using regulated exocytosis and a granular pool of stored sphingolipids [61]. The erythrocyte specific mechanism of S1P secretion relies on ATP-binding cassette transporters (ABC-transporter) [52,56].

**Figure 2:**
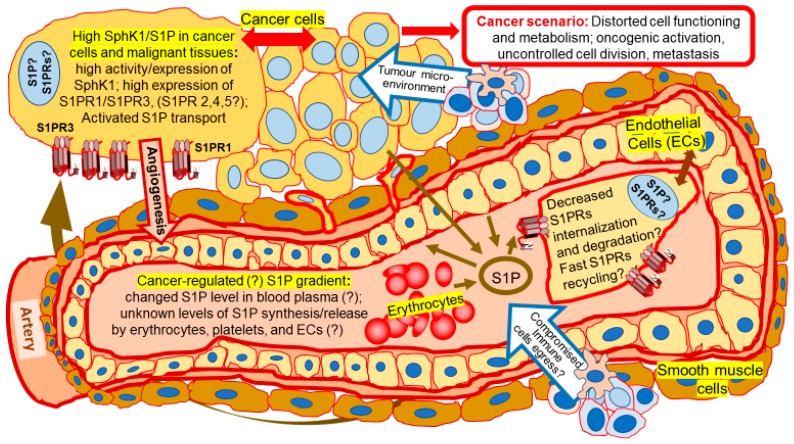
Cancer scenario: Transformation of SphK/S1P/S1PRs signaling. Cancer cells are shown outside of the artery wall. A transverse cut through the artery shows an inside space of the blood vessel filled with blood plasma and blood cells (only erythrocytes are shown), as well as a layer of endothelial cells surrounded by smooth muscle cells. Endothelial cell layer and enlarged single endothelial cell are indicated by a double-ended arrow. Levels of SphK1 expression and activation are high in cancer cells. The regulation of plasma S1P levels and S1P release by blood cells/endothelial cells remain to be confirmed in cancer patients. S1P levels/S1P gradient is hypothetically changed in blood plasma of cancer patients comparing to healthy controls. It is not clear how changed S1P plasma level would influence the level of S1PR expression and signaling. Overactivation and delayed degradation of S1PRs in cancer cells results in reinforced signaling of this pathway and cancer-related pathological consequences including cancer-directed angiogenesis [21,22,23,70,71,72,73,74,75], formation of tumor microenvironment [32,76,77,78], transformed metabolism [68,79], and cancer progression [5,6,11,67,80,81,82,83]. Arrows indicate the movement of S1P and direction of its effects. The arrow directed from S1P to the endothelial cell indicates that S1P also activates endothelial cell signaling via S1PRs. Question marks indicate the gaps in our knowledge of SphK/S1P/S1PRs axis.

Tumor cells were shown to release S1P employing ABC-transporters ABCC1 and ABCG2 after stimulation by estradiol [58] (Figure 2). Suggestively, S1P secretion by endothelial cells, erythrocytes, and platelets might be also controlled by estrogen. Supporting the hypothesis, Guo and co-authors [69] detected an increased level of S1P in the blood plasma of pre-menopausal women compared to post-menopausal women, who have much lower levels of circulating estrogens. The increased S1P synthesis, transport, and expression of S1P transporter proteins ABCC1, G2 and Spns2 were also stimulated by estrogen in the endothelium [69]. That is the first evidence of the potential involvement of estrogen in the regulation of S1P secretion from normal cells.

Released S1P can induce activation of downstream signaling targets via an autocrine and paracrine/endocrine process known as “inside-out” signaling. There are five S1P cell surface G-protein coupled receptors S1PRn (*n* = 1–5) (also defined as endothelial differentiation gene (EDG)) [70,84,85,86]. The receptors were shown to activate the key regulators of growth and survival mechanisms including MAPK [84], Akt/mTOR [70], transcription factor NF-κB [85], Hippo-YAP pathway [87], and Cyclic-AMP Responsive Element Binding Protein CREB [88]. The diversity of S1PR downstream targets was associated with cell type specific expression of the receptors and their differential coupling to a complex network of various G proteins [89]. For instance, the S1PR1 was shown to couple through Gi/o; S1PR2/S1PR3 through Gi/o, Gq, and G12/13; and S1PR4/S1PR5 through Gi/o and G12/13 [89]. Thus, the expression of several S1PR in one cell results in the activation of multiple pathways and creates the opportunity for complex response adjustments.

Various growth factors, hormones, and cytokines induce SphK activation, increase S1P production, and transactivate one or several S1PRs essential for realization of specific intracellular algorithms of these agonists. SphK/S1P signaling was implicated into the maintenance of basic cell functions including cell growth, division, mobility, and signal transduction. Some of these basic functions are also controlled by steroid hormone estrogen, especially in ER-expressing breast mammary cancer cells.

## 3. Estrogen Signaling Network: A Brief Overview

Estrogens control normal cell differentiation and metabolism through all stages of human organism development including the maturation of oocytes and reproductive organs, sex-specific differentiation and the metabolism of many tissues and organs including the brain, heart, lungs, kidneys, intestines, and bones [90]. Later in life, estrogen regulates estrous reproductive cycles and affects sexual and maternal behavior. Estrogens provide lifelong control over the effectiveness of many non-reproductive functions, regulating bone density and strength, blood lipid levels, fat deposition, water and salt balance, and essential brain functions such as memory [1,2,3]. Furthermore, estrogen signaling has important male-specific roles including sperm maturation [90]. Nearly a hundred years ago, it was discovered that estrogens can facilitate breast cancer growth. The detrimental role of estrogen signaling in breast cancer progression was investigated and published in thousands of research articles. For several decades, anti-estrogenic agents were and are successfully used in breast cancer therapy in nearly 70% of all breast cancer patients [91]. However, the search continues for novel anti-cancer agents that would target cancer cell specific mediators of estrogen signaling in therapy-resistant tumors.

Natural steroid hormone estradiol can easily cross a bilipid layer of plasma membrane, penetrate cytosol and nucleus, and bind various intracellular and membrane-associated receptors. The biological activities of estrogen are mediated by specific high-affinity ER including several α- and β-isoforms [92]. ERs act as transcription factors and regulate gene expression [2,3]. Traditional gene-activating effects of the hormone start with the binding of estrogen to ER, which results in the receptor dimerization, nuclear translocation, and the binding of the estrogen-ER complex to an estrogen response element (ERE) and a group of cofactors [93]. Estrogen-bound ERE induces transcription of the tissue specific target genes [2,3,93]. Besides the established genetic activities, accumulating evidence indicates that estrogen can trigger several major cytoplasmic signaling pathways marked by the release of intracellular second messengers such as calcium ions (Ca^2+^) [94], and phosphorylation of mitogen activated protein kinases (MAPK) [1,2,3,13,90]. Membrane associated ER-like hypothetical receptors and G proteins mediate rapid non-genomic estrogen signaling [2,3,13,95]. The estrogen cytoplasmic network incorporates the activation of various pro-survival and mitogenic factors, such as Raf-1 kinase, phosphatidylinositol 3-kinase (PI3K), protein kinase A, and protein kinase C responsible for prominent mitogenic and anti-apoptotic effects of the hormone in breast cancer cells [13,92,93]. Sphingolipids are one of the latest additions to the list of estrogen-induced signaling molecules [5,6,9,10,14].

## 4. Contribution of Sphingolipids in Estrogen and Growth Factor Interactive Effects in Mammary Carcinomas

The link between estrogen and sphingolipids was firstly explored in ER-positive breast cancer cells. To avoid non-specific inhibition, the expression of dominant negative point mutation SphK1 construct (SphKG82D), characterized by the lack of the kinase enzymatic activity, was used as a knock-out tool [31]. Stably and transiently expressed SphKG82D constructs inhibited estrogen-induced growth of MCF-7 breast cancer cells [5]. A time-dependent course of testing uncovered the dual mechanism of regulation: 17β-Estradiol can activate a fast non-genomic and a slower transcriptional course of SphK1 activation. ERα was suggested as the most likely candidate of estrogen-induced SphK1 activation. The leading role of ERα was supported by the absence of estrogen-induced SphK1 activation in ERα-negative MDA-MB-231 cells and in the presence of ERα antagonist ICI_182,780_ [5]. SphK1 inhibition abrogated propagation of estrogen downstream signaling mechanisms including calcium mobilization from intracellular stores and the phosphorylation of Erk1/2 [5]. The key role of SphK in Erk1/2 activation was shown in several other types of cancer cells [71,96,97]. In turn, Erk1/2 phosphorylates SphK1 at Ser225 in HEK293 cells [31].

SphK1 overexpression stimulated proliferation of MCF-7 cells, confirming the kinase strong pro-mitogenic profile [5,6]. Further testing of the biological implementation of SphK1 signaling demonstrated that formation of solid, multilayer, nodular structures (i.e., focus formation) in post-confluent cultures of MCF-7 cells, as well as colony formation in soft agar, were significantly promoted in SphK1 overexpressing cells stimulated with estrogen [5]. Interestingly, estrogen not only increased intracellular levels of S1P, but also stimulated the rapid release of S1P and dihydro-S1P from MCF-7 cells. The estrogen-induced export of S1P was blocked by specific inhibition of ABCC1 (multidrug resistant protein 1) or ABCG2 (breast cancer resistance protein) transporters [58].

Increased division and proliferation of several normal and cancerous tissues was marked by SphK1 activation and increased S1P production. SphK1 was activated by various growth factors including insulin-like growth factor (IGF) [98,99], platelet-derived growth factor (PDGF) [100], vascular endothelial growth factor (VEGF) [71], and epidermal growth factor (EGF) [14,101]. Glucose metabolism-regulating hormone Insulin was also shown to activate the SphK pathway. Notably, both SphK isoenzymes were equally responsible for insulin-induced cell cycle progression and the proliferation of MCF-7 breast cancer cells, although SphK1 and SphK2 had different roles in mediating insulin-induced ERK1/2 and Akt activation [102]. The triggering of the kinase by hormones and growth factors provides required sphingolipid turnover, which is essential for membrane reconstruction after the initiation of growth and division. Moreover, several cancer types demonstrated continuously increased levels of SphK1 expression [10,103], confirming selective requirement for the presence of this kinase in the maintenance of malignancy.

The positive feedback loop that exists between the sphingolipids and growth factor signaling network can amplify the survival and proliferative capacities of cancer cells. SphK1 product S1P can initiate tyrosine phosphorylation of EGFR in breast cancer cells [14], which was consistent with the observations previously reported in vascular smooth muscle cells [104] and fibroblasts [105]. Specifically, SphK1 and S1PR3 mediate the estrogen-induced EGFR transactivation in ER-positive breast cancer cells [14]. Moreover, S1PR3 was important for insulin-mediated mitogenic action in breast cancer cells [102]. The interaction of EGFR with estrogen signaling can occur by the tripartite mechanism that heavily relies on the sphingolipid signaling pathway. The ERα and GPR30 mediated the tripartite pathway’s cross-talk. The evidence was generated using antisense oligonucleotides for GPR30, while ICI_182,780_ was used to block ERα signaling [14].

The involvement of ERβ in activating SphK/S1P signaling was not properly tested in mammary cancer cells. However, one of the recent investigations partially addressed this question and tested the effects of the phytoestrogenic compound genistein, the most prominent isoflavone from soy in epithelial keratinocyte cells. The study indicated the primary role of ERβ in activation of SphK1 and S1P production by genistein [106]. The phytoestrogen treatment stimulated acidic and alkaline ceramidase expression and cellular S1P levels. Inhibition of S1P lyase was also increased. The authors suggested that activation of ERβ and S1P production might be linked to antimicrobial effects of the phytoestrogen upstream of NF-κB signaling as part of the epithelial innate immunity defense [106]. Complete receptor knock-out and/or use of KO mice should clarify the uncertainties of ER type involvement in sphingolipid signaling in future studies.

It is important to note that the estrogen-initiated SphK1-mediated EGFR transactivation also involved matrix metalloproteases (MMP) and c-Src, as the signaling was inhibited in the presence of Src-specific inhibitor PP2 and MMP inhibitors *o*-phenanthroline or GM6001 [14]. Interaction between Src and SphK1 pathways was detected in several other malignant and normal cell types. In ERα-negative breast cancer cell lines MDA-MB-231 and BT-549 Src, family kinases were linked to leptin-induced SphK1 effects [107]. Central nervous system-infecting *E. coli* was shown to exploit S1P for EGFR activation and facilitate bacterial penetration of the blood brain barrier through downstream Src triggering [108]. Src also mediated some SphK2 effects [109]. Total Src protein levels were significantly decreased in Sphk2^−/−^ megakaryocytes. Both SphK2 and Src deficiency resulted in defective intravascular proplatelet shedding, the final stage of thrombopoiesis [109]. The impact of SphK1 on Src expression has not been tested. Noteworthy, the ERα and cSrc binding was detected previously [110], suggesting that a potentially mediatory and/or adaptor-like role of cSrc for ERα/SphK1 interactions should be addressed in future studies.

## 5. Role of Sphingolipids in Development of Drug Resistance

Various drug-resistant cells demonstrated higher levels of SphK1 expression *in vitro*. For instance, imatinib-resistant K562 cells were marked by upregulated SphK1 responsible for the resistant phenotype [111]. SphK1 was also overexpressed in temozolomide-resistant glioma cells [45]. Recently, the resistance-promoting role of SphK1 was confirmed by clinical investigations *in vivo*. Higher SphK1 expression was detected in several types of advanced and aggressive tumors by a prospective neoadjuvant chemotherapy trial [112]. An increased pathological complete response rate was also observed in tumors with high SphK1 expression within the luminal mammary cancer subtype group [112].

Elevated levels of SphK1 expression and activity coincided with increased EGFR protein level in tamoxifen resistant MCF-7 cells [9]. Responsiveness to tamoxifen itself inversely correlated with the levels of SphK1 expression and activity. Employing various *in vitro* strategies, it was demonstrated that SphK1 regulates sensitivity to tamoxifen in parental MCF-7 cells and the tamoxifen-resistant cell line. High levels of intracellular SphK1 counteracted tamoxifen-induced apoptosis [11]. On the contrary, inhibited or down-regulated levels of SphK1 elevated the pro-apoptotic effects of tamoxifen, thus restoring tamoxifen toxicity [9]. Furthermore, specific SphK inhibitors SKI-II and ABC294640 exaggerated radiotherapy- and/or chemotherapy-induced cell death, and helped to reduce drug resistance [11,12,113]. SKI-II dose-dependently decreased the estrogen-stimulated estrogen response element (ERE) transcriptional activity and diminished mRNA levels of the ER-regulated genes progesterone receptor and steroid derived factor-1 (SDF-1). The inhibitor could directly bind the ER in the antagonist ligand-binding domain, thus acting as a novel ER-signaling blockade in breast carcinoma [113].

Cancer resistance theory declares that tumors originally responsive to chemotherapy treatment might contain a group of cells able to endure and outlive the hostile chemotherapy/radiotherapy environment. After an undefined quiescent period, these cells commence proliferation, acquiring a resistant phenotype. Malignant cells with an advanced resistant phenotype and aggressiveness were also marked by a distinctive expression of S1PRs. In a cohort of 304 ER-positive breast cancer patients, high membrane S1PR1 expression was correlated with shorter time to recurrence, while high cytoplasmic S1PR1 and S1PR3 were linked to shorter disease-specific survival times [10]. Moreover, Ohotski and colleagues [35] demonstrated that nuclear localization of SphK1 along with either ERK-1/2, or SFK, LYN, AKT, NF-κB reduces disease-specific survival and recurrence. The same research group found that nuclear presence of S1PR2 proteins was associated with improved prognosis. Thus, a cancer promoting role was suggested for S1PR1 and S1PR3 subtypes [9,10,14,18,86], while S1PR2 presence demonstrated potential growth-limiting effects [35,72,114,115]. It is not unusual to observe the increased expression of S1PR3, although the S1PR1 is often lost or decreased in many cancer cells [116,117,118]. Change of the type of S1PR expression and/or expression ratio often follows the shift from normal towards malignant transformation, and further towards a more advanced and metastatic cancer phenotype [116,117].

Recently, we tested the regulation of S1PRs expression by widely used pro-apoptotic chemotherapy drugs in MCF-7 cells [62]. We detected that tamoxifen (TAM) and/or Medroxy-progesterone acetate (MPA) downregulate expression of both mRNA and protein S1PR3 levels. In contrast with S1PR3, levels of S1PR2 were increased in MCF-7 cells treated with TAM/MPA. Interestingly, tamoxifen-induced autophagy and apoptosis coincided with the S1PR2/S1PR3 ratio changes [62]. However, the exact mechanisms of S1PR regulation and its association with the stimulation of cell death remain to be further investigated.

The therapeutic potential of sphingolipids has been recently assessed in ER-negative mammary cancers. Hait et al. [119] tested the anticancer actions of FTY720 (Fingolimod, Gilenya), a sphingosine analog, the Food and Drug Administration (FDA)-approved prodrug for treatment of multiple sclerosis. Oral administration of FTY720 inhibited development and progression of spontaneous breast tumors *in vivo*. However, the most surprising effect of FTY720 was the re-expression of estrogen and progesterone receptors in previously triple negative tumors. Furthermore, FTY720-reactivated expression of silenced ERα sensitized these tumors to TAM-induced apoptosis [119].

The overexpression of growth factor receptor and their administrative role in the development of chemoresistance have been widely observed in cancer cells [120]. Different forms of mammary, lung, and brain cancers were strongly associated with increased expression, mutation, and/or hyperactivation of EGFR [121]. To avoid prolonged signaling, activated EGFR should be removed from plasma membrane (internalized) and directed for degradation (endocytosis) [122]. Interruption of this mechanism would potentially result in uncontrollable growth stimulation. We found that EGFR levels were increased in the SphK1-overexpressing cancer cells [9]. Estrogen/S1P treatment of MCF-7 cells resulted in accumulation of EGFR in endosomal compartments, while the membrane EGFR was quickly internalized [17]. Interestingly, both estrogen and S1P also activated cdc42, a member of Rho family of small GTPases and upstream inhibitor of EGFR ubiquitination and degradation [123]. Further analysis confirmed that inhibition of EGFR degradation and associated enhanced EGFR level are caused by estrogen/S1P-induced stimulation of cdc42 signaling [17]. This mechanism further defines a novel cancer-related route of ligand-independent regulation of EGFR endocytosis, recycling, and degradation by estrogen/S1P.

An interaction between human EGF receptor 2 (HER2) and SphK1 was assessed in a few studies [124,125,126,127]. Both HER2 and SphK1 can stimulate activation of growth and survival effectors, including MAPK and Akt. However, SphK1 signaling was suppressed in HER2 positive breast cancer, likely by a negative feedback mechanism that favors only one survival and proliferation enhancing pathway. Supporting this suggestion, S1P levels were lower in cancer patients with HER2 overexpression and five assessed patients with HER2 overexpression/amplification demonstrated negative expression of pSphK1 [126]. Alternatively, S1P levels were higher in HER2 negative cancers [126]. Furthermore, HER2-negative ER+ breast tumors with high SphK1 expression demonstrated resistance to chemotherapy and poor prognosis [127]. Unexpectedly, patients with both ER+ HER2-positive tumors and high SphK1 were characterized by improved survival and reduced tumor recurrence after tamoxifen treatment [124]. The detected clinical data suggest pathway interactions, potentially of a mutually-exclusive capacity among ERs, SphK1, and HER2-signaling in breast cancer cells. Although these findings remain controversial, as another group reported that high expression of SphK1 in ER-negative HER2-positive tumors was associated with reduced disease-free survival [125]. SphK1 was also shown to stimulate HER2-positive breast cancer development through increased claudin-2 expression [127]. The low number of tested samples and controversial findings indicate a requirement for more detailed testing of HER2-SphK1-ER cross-talk.

## 6. S1P Signaling in Stem Cells

The regulatory role of sphingolipids has been assessed in several types of precursor pluripotent cells. One of the first reports indicated that S1P signaling influenced cell division responses of myogenic precursor cells. Distinct S1PR subtypes were linked to promotion of skeletal myoblast proliferation, differentiation, and survival [128,129,130,131], as well as satellite cell growth [132,133]. S1P signaling was also implicated in regulation of muscular resistance to injury and apoptosis [132]. Exogenously secreted S1P mediated preservation of satellite cell viability and renewal [132], indicating potential participation of S1PRs in the functioning of this type of progenitor cells. Locally, specific S1PRs could be activated in autocrine or paracrine manner via S1P production. Bone-marrow-derived mesenchymal stromal cells (MSCs) were shown to synthesize and release a large amount of S1P that was required for MSC-mediated effects on muscle C2C12 myoblasts and satellite cell proliferation [134]. Besides growth regulation, the differentiation-related role of S1P signaling was shown during analysis of transforming growth factor β (TGFβ) effects in myoblasts. Specifically, S1PR3 directed TGFβ-linked myoblast trans-differentiation towards fibrosis rather than myogenic differentiation [135].

Proliferative and chemotactic effects of VEGF signaling were also mediated by SphK/S1P in muscle progenitor cells [134]. However, the mutual interaction between S1P and VEGF networks is not exclusive to progenitor cells. The role of SphK/S1P axis as VEGF mediator was previously described in several types of normal [38,104,136] and malignant cells [71,96,137,138]. VEGF was shown to stimulate S1PR1 expression [38,70,136], and increase activation of SphK and S1P production [71,137]. Moreover, VEGF Receptor-2 (VEGFR-2) was shown to form signaling complex with S1PR1 [96,137]. In turn, S1P stimulated VEGF expression and transactivation of VEGFR-2 [104,136,137,138].

Circulation of lymphoid progenitors is orchestrated by the S1P/S1PR axes [139]. S1PRs expressed on the surface of hemopoietic progenitors are highly sensitive to S1P exposure that activates rapid internalization of the receptors. The mechanism seems essential for the egress of progenitor cells from bone marrow to peripheral blood. Notably, not only S1P, but also C1P enhanced migration of endothelial and lymphoid progenitor cells [140]. S1PR1 and S1PR3 axes were shown as dominating mediators of S1P responses in endothelial progenitor cells (EPCs). In patient-derived EPCs, the activation of CXCR4 signaling was triggered by the S1P/S1PR3 network [141]. Similarly, besides EPCs, S1PR1 and S1PR3 activation was shown to regulate mobility and migration of malignant cells [116,142].

S1PR1 was suggested as the major S1PR expressed in the Kit(+)/Sca-1(+)/Lin(−) (KSL) hematopoietic stem progenitor cells (KSL-HSPCs). Notably, the selective S1PR1 antagonist W146 significantly stimulated KSL-HSPC mobilization from bone marrow into peripheral blood in mice. The potential inhibition of migration was exclusive to S1PR1, suggesting the positive role of the receptor in the retention of hematopoietic cells in a bone marrow microenvironment [143]. Beyond the egress-inhibiting role of S1PR1, the receptor role was critical in the stimulation of adipose-derived stem cell proliferation [144], the revascularization of endothelial colony-forming cells [145], and the promotion of vascular endothelial progenitor cell angiogenic activity assessed by proliferation, wound healing, 3D sprouting, and directed migration assays [73]. Notably, not the growth-promoting, but rather the differentiation-related role of S1P was shown in C3H10T1/2 multipotent mesenchymal stem cells, where S1P promoted osteogenic and inhibited adipogenic differentiation of the progenitor cells [146]. Further down in this review, we will discuss the recently discovered roles of sphingolipids in the regulation of adiposity and related metabolic syndrome.

The expression of S1PR1 in a hypothetical endothelial and hemopoietic precursor was also required for appropriate morphogenesis of the kidney vasculature, including glomerular capillary development, arterial mural cell coating, and lymphatic vessel development [147]. Altogether, S1PR1 and S1PR3 were often positively involved in regulating capillary-like formation and migration, while S1PR2 exerted various effects including a negative role on the migratory capacity of mesenchymal progenitor mesangioblasts. In these cells, SphK inhibition and/or S1PR1/S1PR3 down-regulation significantly reduced *in vivo* angiogenesis [74]. Contrary to this, proliferation of small hepatocyte-progenitors and stem (oval) cells during liver injury was clearly associated with S1PR2 and S1PR4 expression [148]. In primary CD34^+^ mononuclear cells obtained from chronic myeloid leukemia (CML) patients, molecular or pharmacologic inhibition of SphK1/S1PR2 signaling enhanced imatinib- or nilotinib-induced growth inhibition [149], indicating the proliferation-promoting role of the sphingolipid network and specifically S1PR2 in these type of hemopoietic progenitor cells.

A heterogeneous expression pattern of S1PR subtypes was observed between muscle stem and fully differentiated muscle cells. For instance, S1PR1 was predominantly expressed in cardiomyocytes, while S1PR2 and S1PR3 receptors were detected in cardiac progenitor cells (CPCs) [150]. Activated by the S1PRs, Rho signaling mediated the proliferation of CPCs. Unexpectedly, both receptor subtypes S1PR2 and S1PR3 were shown to regulate RhoA activation through Gα12/13 while mending myocardial injuries [150]. S1PR2 was essential for proper endoderm formation and CPC migration during the building of the primary heart tube in zebrafish [151]. Previously, S1PR2 anti-proliferative effects were observed in various types of cells [62,114,152]. Contradicting the found effects in progenitor cells, the inhibition of Rac and related cell migration were among the S1PR2-regulated responses [153]. Considering the high specialization of stem cells, S1PR2 might be serving specific pluripotency supporting purposes. However, Rho signaling that mediated S1P effects in non-pluripotent cells [114] was also activated in CPCs [72,150,153], thus indicating that S1PR isoforms are linked to the similar downstream effectors independently of pluripotency. Exactly how, despite similar downstream effectors, the S1PRs induce different effects in normal, malignant, and progenitor cells is a puzzle which remains to be solved.

Therapeutic S1PR agonist/antagonists were tested in pluripotent multilineage hemopoietic cells. It was shown that potent S1PR antagonist FTY720 increases the chemotactic responsiveness and migration of human CD34^+^ lineage-committed progenitor cells for mixed lineages, granulocyte-monocytes, and erythroid cells [154]. Furthermore, FTY720 increased the viability and neurogenicity of irradiated neural stem cells from the hippocampus [155]. FTY720 caused a remarkable cytoskeletal change with deformed and decreased filopodia formation, decreased the ability of cancer cells to adhere and migrate, reduced the expression of integrins, induced apoptosis, and prevented metastasis in breast cancer cells [156]. FTY720 reactivated expression of silenced ERα and sensitized them to tamoxifen [119]. These diverse multifunctional effects of FTY720 suggest an ability of this agent to interact with more than one specific target in tested cells. Thus, the exact mechanisms of FTY720 signaling remains to be tested in breast cancer stem cells (CSCs).

However, some effects of the S1P network were recently explored in CSCs models [18,19]. The stimulation of pluripotency by sphingolipids was addressed after treatment of breast cancer cells with environmental carcinogens including toxic phthalate and benzyl butyl phthalate (BBP). These agents activated aryl hydrocarbon receptor that, in turn, stimulated SphK1/S1P/S1PR3 signaling and enhanced the formation of metastasis-initiating breast CSCs. The knockdown of SphK1/S1PR3 effectively reduced tumor growth and lung metastasis *in vivo* [19]. Furthermore, S1P enhanced the mammosphere-forming capacity of aldehyde dehydrogenase (ALDH)-positive CSCs via S1PR3 and subsequent activation of the Notch signaling pathway. SphK1-overexpressing CSCs demonstrated an increased ability to develop tumors in nude mice, while tumorigenicity of these CSCs was also inhibited by S1PR3 knockdown or S1PR3 antagonist [18]. PCR assays indicated a high expression of S1PR3, but lower S1PR2 in the ALDH-positive CSCs population [18]. Further experimental work is required to uncover the specific role and mechanism of SphK/S1PRs signaling axis in progenitor cells.

## 7. Is SphK Intracellular Localization Connected to Its Functions?

SphK1 was first reported to localize mostly in cytosol where ERK1/2-dependent activation and phosphorylation of SphK induced membrane translocation of the enzyme [31]. This membrane translocation of SphK1 was defined as a critical determinant of cancer progression [8,31]. Intracellular S1P was initially shown to mediate calcium release from intracellular stores [157], but a target ion channel or transporting protein has not been unequivocally identified. Both SphK subtypes were also detected in nuclear space, suggesting that enzymes can potentially influence genomic signaling [35,38,119,158]. For instance, SphK2 has a nuclear localization sequence, can localize in the nuclei, and cause inhibition of DNA synthesis and other anti-proliferative effects [30,38]. The enzyme’s nuclear localization pattern requires protein kinase D-mediated phosphorylation for translocation to cell nucleus [37]. However, SphK2 also generates S1P in cytoplasm, and specifically in the endoplasmic reticulum. It was suggested that the opposite effects of SphK1 vs SphK2 on cell proliferation and G1/S transition are dependent on the enzymes localization. Thus, targeting SphK1 to the nucleus was supposed to mimic the DNA synthesis inhibitory effect of SphK2 [38]. However, this hypothesis remains controversial. Immunohistochemistry (IHC) analysis of ER-positive mammary tumors indicated that nuclear SphK1 can co-localize with ERK-1/2, LYN, AKT, or NF-κB within the space of the nuclear envelope. Further analysis demonstrated that nuclear presence of SphK1 is largely detrimental and significantly shortens disease-specific survival and accelerates cancer recurrence [35]. The authors also discovered that presence of nuclear S1PR2 and Src is associated with improved prognosis linked to a reduction of SphK1 [35].

Histone deacetylases (HDACs) are the recently discovered nuclear S1P targets. Following the specific activation of SphK2, generated S1P binds and inhibits HDACs, the factor which modulates the dynamic balance of histone acetylation and provides the epigenetic regulation of specific target genes [119,158]. Furthermore, FTY720, the sphingosine analog, can be phosphorylated and activated by nuclear SphK2 and accumulates in the nuclear space. The nuclear phosphorylated FTY720 is not only a potent inhibitor of HDACs, but also regulates expression of a restricted set of genes [119]. Besides HDACs, S1P produced by SphK2, binds to prohibitin 2 and assists in the regulation of the respiratory complex IV assembly to control respiration in mitochondria [159]. S1P, which is also generated by SphK2, can bind human telomerase reverse transcriptase (hTERT) at the nuclear periphery in human and mouse fibroblasts [160]. Notably, SphK2 downregulation or mutations of the S1P binding site decreased the stability of hTERT in cultured cells and promoted senescence via loss of telomere integrity. Conclusively, S1P binding to hTERT promotes telomerase stability and facilitates tumor growth [160]. On the contrary, other authors demonstrated that SphK2 expression causes cells to arrest in G1/S [38]. Considering other genomic signaling mechanisms, SphK1 was shown to bind TRAF2 [63]. Produced S1P influenced E3 ubiquitin ligase activity of TRAF2, an essential mediator of the nuclear factor-κB (NF-κB) pathway and TNFα signaling network [155,158]. Both HDAC1/2 and NF-κB intracellular pathways were suggested to serve as S1P downstream targets linked to cancer initiation and progression [63,158,161,162]. However, it is still largely unclear how SphK1 is transported through the nuclear envelope. Mechanisms of SphK1 signaling in the nucleus are also unclear.

The intracellular localization of S1PRs was recently tested by several research groups. Wang and colleagues [36] using tissue IHC microarrays analyzed 384 formalin-fixed paraffin-embedded blocks containing 183 benign and 201 different malignant tissues. It was found that all five S1PRs are widely distributed in multiple human organs and systems. S1PRs were expressed in both the cytoplasm and nucleus, except for S1PR3 which was seen in the nucleus only. Surprisingly, the S1PRs were rarely found on cellular membranes even though S1PRs are a transmembrane G-protein coupled receptor type [86]. S1PR subtypes were distributed in nuclear and/or cytoplasmic compartments in a tissue specific manner. Strong nuclear localization was demonstrated for S1PR1, S1PR3, and S1PR5, whereas S1PR2 and S1PR4 show only weak staining. The adrenal gland, liver, brain, and colon were marked by significant differences in IHC scores for the multiple S1PRs (nuclear and/or cytoplasmic). Stomach, lymphoid tissues, lung, ovary, cervix, pancreas, skin, soft tissues, and uterus IHC demonstrated differences for only one S1PR (cytoplasmic or nuclear). However, overall, 23 organs/tissues had no significant difference in IHC scores for any S1PR (cytoplasmic or nuclear) localization between benign and malignant transformation [36].

We have previously reported membrane and cytosolic localization of S1PR3 in MCF-7 breast cancer cells [9,17]. We have also observed nuclear localization of S1PR1 in nuclear space of endothelial cells treated with Estrogen or S1P [21]. Considering demonstrated previously estrogen-dependent activation of Erk1/2 [13] and SphK1 [5,6], it is logical to suggest a possibility of SphK1 phosphorylation and membrane translocation. However, the question remains untested whether estrogen can induce translocation of SphK to plasma membrane or to nuclear space in cancer and/or normal cells.

Previously, nuclear localization of ectopically-expressed wild type S1PR2 was associated with induction of apoptotic gene expression in ER-negative MDA-MB-231 cells by an unknown mechanism [35]. Even though G-protein coupled receptors (GPCRs) are supposed to sense the extracellular changes, several GPCRs were recently found in the nuclear space, suggesting novel roles for these receptors beyond those traditionally established [163]. For instance, distinct but complementary angiogenic roles of F2rl1 (formerly known as PAR2) showed that it was dependent on its subcellular localization at the plasma membrane or at the nucleus [163]. However, specific physiological functions of nuclear S1PRs remain to be uncovered. 

## 8. Involvement of SphK/S1P in the Regulation of Apoptosis and Autophagy

The most studied sphingolipids in terms of their roles in regulation of programmed cell death are ceramide (Cer), sphingosine (Sph), and S1P [164,165,166]. While SphK1 and its product S1P are potent anti-apoptotic effectors, Cer and Sph were shown to initiate and promote several types of cell death [118]. S1P and Cer balance is regulated by SphK1 via a mechanism called sphingolipid rheostat that was shown to play a key role in directing cellular responses to oxidative stress. While Cer is increased within the cell during oxidative stress and senescence, activation of SphK1 and increased levels of S1P can abrogate and reverse that process [118,166]. Extreme stress eventually results in SphK1 degradation and decreased levels of S1P, thus shifting the rheostat balance toward increased levels of Cer/Sph and cell death [118].

SphK/S1P anti-apoptotic effects were detected in many types of cells stimulated with strong pro-apoptotic stimuli including Fas ligands, cytokines, anti-cancer drugs, serum and growth factor deprivation, and ionizing radiation [8,64,118,167]. Considering the most established effects, SphK/S1P and S1PRs block or diminish caspase activation (including caspases 3, 6, and 7) [66,167,168], inhibit stress-activated protein kinase JNK [169], and change the ratio between pro- and anti-apoptotic members of the Bcl2 family towards pro-survival molecules [8,45,166]. Under condition of severe hypoxia, SphK1 promotes the Warburg effect, the cancer associated metabolic adaptation to low oxygen condition that is the essential survival mechanism for cancer cells to produce ATP and protect against reactive oxygen species (ROS) [79]. Furthermore, transactivation of growth factor signaling network [14], phosphorylation and activation of Erk1/2 [31], and PI3K/Akt [8,21,135] are among the pro-survival molecular machinery employed by SphK/S1P signaling. Many of these mechanisms are also triggered by estrogen and the ER cytoplasmic pathway [64,65].

The anti-apoptotic effects of SphK/S1P/S1PRs were registered in different cancers including ER-positive breast cancer cells [167,168]. It has been shown that overexpressing SphK MCF-7 cells are more resistant to programmed death induced by the anti-neoplastic agent doxorubicin and TNFα than the control parental cells [11,12,113]. Since doxorubicin stimulates the accumulation of Sph that triggers mitochondria-triggered apoptosis, transformation of Sph by SphK1 should deliver an anti-apoptotic effect. Accordingly, increased expression of SphK1 was linked to inhibition of caspase-7 and poly(ADP-ribose) polymerase (PARP), resulting in enhanced survival capacity [168]. Tamoxifen resistance was also marked by increased levels of SphK1 in MCF-7 cells [9].

Autophagy is another specific type of cell death that was recently shown to be regulated by sphingolipids. Autophagy is defined as the degradation and utilization of cellular components in specialized organelles called autophagosomes that are designated to fuse with lysosomes during normal cell component recycling [170], nutrient depletion, and/or other resource-limiting stress [171]. The role of autophagy is controversial. It can serve as a survival pathway for cells that require elimination of damaged organelles or utilization of excess resources under pressure of a limited nutrients supply. Alternatively, when stress exceeds the tolerance threshold, autophagy causes cell death [171]. SphK1 overexpression and nutrient starvation induced autophagy in MCF-7 cells and was associated with cell survival [172]. Decreased activity of the mammalian target of rapamycin (mTOR) and a modest increase in Beclin-1 mediated the SphK1-induced autophagy [172]. The increased level of Beclin-1 coincided with changed levels of S1PR2 and S1PR3 during tamoxifen- and/or medroxy progesterone acetate (MPA)-induced autophagy in MCF-7 cells [62]. Tamoxifen or MPA treatment induced downregulation of S1PR3, but stimulated expression of S1PR2 along with the activation of both autophagy and apoptosis pathways [62]. 

Interestingly, the knockdown of a specific phosphatase that degrades S1P, sphingosine-1-phosphohydrolase-1 (SPP1), can also trigger a pro-survival type of autophagy. In contrast to SphK1 overexpression, autophagy induced by SPP1 downregulation was marked by the activation of endoplasmic reticulum stress and the unfolded protein response, but did not change Beclin-1 or mTOR signaling [173]. Activation of the unfolded protein response sensors PERK and PI3K resulted in increased pAkt, an important determinant of cell survival within S1P-induced autophagy [173].

The involvement of SphK/S1P in the regulation of autophagy-related signaling was recently demonstrated in ovarian cancer cells [67]. FTY720, an immunosuppressant that can bind SphK and S1PRs, demonstrated strong cytotoxic effects in human ovarian cancer cells resistant to cisplatin [67]. The FTY720 treatment also increased the number of autophagosomes, accumulation of LC3-II, and p62 degradation. Necrotic response to FTY720 was aggravated by the blockade of autophagy, supporting the theory that inhibition of autophagy could augment anticancer drug potency [67].

## 9. Regulation of Cell Shape, Mobility, and Metastasis by Sphingolipids

Sphingolipids were shown to direct cell migration and mobility, metastasis, angiogenesis, epithelial to mesenchymal transition (EMT), and immune cell trafficking. S1P and ceramide-1-phosphate (C1P) are potent chemoattractants for a variety of cells including stem/progenitor cells [174]. Released from dying and/or injured cells, S1P also attracts macrophages to the damaged area [76]. Similarly, to attract immune cells, S1P levels are high in blood and lymph, but low in tissues (Figure 1). The difference in concentrations creates a gradient of S1P that is important for vascular integrity and egress of immune cells from lymphoid organs to circulation [77]. Inflammatory processes and injuries stimulate the production and release of S1P, which in turn facilitates the recruitment of immune cells to the damaged and/or inflamed area [78]. The S1P/S1PR1 axis promotes endothelial precursor recruitment and vascular development during embryonic development [75] and tumor progression [23]. For instance, lymphocyte migration is regulated by FTY720, which triggers S1PR1 endocytosis and degradation. Forced elimination of S1PR1 prevents lymphocyte egress and diminishes inflammation [175].

The migration of several cancer cells was promoted by S1PR1/3 [7,9,124,166], but prevented by S1PR2 [176]. For instance, S1P-stimulated migration of melanoma cells was facilitated by the expression of S1PR1, but inhibited by S1PR2 [117,177]. The ratio of S1PR3/2 expression was also critical for the migration of gastric cancer cells [116]. Nevertheless, the role of S1PR2 in the regulation of migration remains controversial, as few reports have demonstrated the stimulation of migration by the receptor [178].

Down-regulation of SphK1 was shown to reduce S1PR3 expression and associated ERK-1/2 activation and migration of MCF-7 cells [124]. Accordingly, it was suggested that SphK1 can regulate cell responsiveness to S1P by increasing S1PR3 expression levels, thus producing a positive amplification loop of S1PR3-mediated invasive signaling in breast cancer cells [10,35,86]. S1PR3 promoted migration and mobility not only of cancer cells [9,179], but also of normal cells, including bone marrow-derived mesenchymal stem cells [180]. The role of S1PR1 and -3 was also positive in the activation of angiogenesis and migration of endothelial cells [21,54,70]. High expression of SphK1 and increased S1P levels were associated with lymphatic metastasis in breast cancer tissue [126]. Upregulation of S1PR3 was recently detected in analysis of metastatic breast cancer lines [181]. Altogether, increased metastatic capacity was linked to the SphK/S1P signaling network in many cancers including hepatocellular carcinoma [182], pancreatic [80], ovarian [183], colorectal [81], gastric [82], and esophageal cancer cells [83]. The metastasis-facilitating property of SphK/S1P axis represents an attractive therapeutical target that can be potentially fixed by one group of agents in different cancers. 

Plasma membrane reconstruction and flexibility are always observed during cell movement and metastasis. Sphingolipids are involved in movement-associated membrane transformation such as filopodia and lamellipodia formation, as well as cell shape change, including EMT. The mechanism of cell shape transformation by sphingolipids was associated with S1P signaling and the S1PRs expression profile. Specifically, the filopodia formation was stimulated by S1P via phosphorylation of the ezrin/radixin/moesin (ERM) family of cytoskeletal proteins in HeLa cells transfected with recombinant bacterial sphingomyelinase [184]. Further experiments indicated that S1P/S1PR2 induced cytoskeletal rearrangements, phosphorylation of ERM proteins, and subsequent filopodia formation and invasion [185]. S1PR2 also mediated EGF-dependent activation of invasion [186]. The novel mechanism of S1P-mediated invasion was recently shown in a cancer xenograft model *in vivo*. Endogenously produced and extracellular S1P upregulated expression of C-reactive proteins (CRP) in breast carcinoma cells [187]. CRP expression was crucial for the transcriptional activation of matrix metalloproteinase-9 through Erk, ROS, and c-fos, leading to breast cell invasion under inflammatory conditions [187].

Besides cancer cells, S1P is a potent chemoattractant for endothelial cells. S1P stimulated cortactin translocation to the cell periphery to form lamellipodia and regulate membrane ruffling and endothelial migration/chemotaxis. In contrast to filopodia, the process was mediated by S1PR1 and Cdc42/Rac activation pathways [188]. S1PR1 was suggested to integrate signals from platelet-derived growth factor (PDGF) receptor to lamellipodia extension and vascular cell migration [100]. PDGF-induced focal adhesion formation and activation of FAK/Src, stress-activated protein kinase 2, and p38 were mediated by S1PR1 in fibroblasts. The process was initiated by the recruitment of SphK1 to the cell’s leading edge, followed by localized formation of S1P. Spatial and temporal stimulation of S1PR1 was essential for directional movement of cells toward chemoattractants [100]. 

However, S1P effects are tissue specific and were shown to block cell migration in some studies. For instance, S1PR2 negatively regulated migratory responses of vascular endothelial cells [189]. S1P inhibited pseudopodium formation by blocking polymerization and reorganization of actin filaments in newly formed pseudopodia, and reduced F-Actin by approximately 25% in mouse melanoma cells. The same study demonstrated that S1P can interact with molecules associated with actin nucleation to inhibit the reorganization of pseudopodium formation and cell mobility [190]. Furthermore, esophageal S1PR5 over-expressing Eca109 cells, although displaying spindle cell morphology with elongated and extended filopodia-like projections, responded differently to S1P treatments. Contrary to the finding, which demonstrated a positive S1P effect on cell mobility [184,185], S1P binding to S1PR5 inhibits the proliferation and migration of Eca109 cells. Thus, it was hypothesized that the expression of S1PR5 is down-regulated to avoid the inhibitory effect of S1P in esophageal cells [83]. 

Cell movement is often associated with the change of cell shape. The mechanism of rapid cell retraction induced by S1P was associated with a quick spike of activated RhoA and a coalescence of adhesion complexes that colocalize with the ends of stress fibers in NIH3T3 clone7 cells in vitro [191]. The study indicated that S1P-induced retraction required RhoA and ROCK, and that pseudopodia sense and integrate signals initiated by localized bioactive sphingolipids, affecting both cellular polarity and mobility [191]. S1P treatment was also shown to activate the Src family kinase that triggers recruitment of the F-actin-binding protein cortactin to sites of actin polymerization at the rim of membrane ruffles. Both Src and Rac pathways are essential for lamellipodia targeting of cortactin. Further, Src plays a determinant role in S1P-induced cell spreading and migration. Src- and Rac-dependent events couple S1P receptors to the actin polymerizing machinery that drives the extension of lamellipodia and cell migration [20]. Both SphK1 and SphK2 were shown to initiate cell shape transformation. For instance, SphK2 isoform activation promoted actin rearrangement into membrane ruffles/lamellipodia in response to S1P in MCF-7 breast cancer cells [192]. Filamin A, an actin cross-linking molecule important for cell movement, was detected as a bona fide SphK1-interacting protein. SphK1 was further required for heregulin-induced migration, lamellipodia formation, activation of PAK1, and subsequent Filamin A phosphorylation [15,16,166].

S1P also contributes to the activation of EMT when epithelial cells develop invasive mesenchymal cell characteristics associated with tumor progression. EMT could be induced by growth factors, cytokines, and matrix metalloproteinases (MMPs). Increased evidence has demonstrated that the cell surface glycocalyx is closely associated with S1P and plays an important role in tumor progression, suggesting that S1P-induced EMT could be Snail-MMP signaling-dependent [182]. Considering the activation of SphK/S1PR signaling by estrogen, it is important to mention that estrogen was also shown to stimulate EMT in breast cancer cells [193,194]. However, it was not tested whether S1P/SphK/S1PR mediate estrogen-induced EMT in cancer cells.

## 10. Sphingolipid Signaling in Terminally Differentiated Cells: What Can Be Used in Cancer Prevention?

Besides established growth- and apoptosis-related effects in cancer cells, sphingolipids were shown to regulate specific biological functions of various non-malignant terminally differentiated cells. Among the most investigated are cells of the immune and circulatory systems. Erythrocytes, platelets, and endothelial cells were defined as the main source of plasma S1P that is produced and released by these cells in abundance [50,52,54,56,61,195] (Figure 1). Thus, a high level of S1P production and storage does not necessarily result in or facilitate malignant transformation and stimulate uncontrollable cell division. As platelets and erythrocytes have no nuclei, endothelial cells should be more closely investigated for the mechanisms of adaptation to high levels of sphingolipid metabolism and S1P synthesis that were shown to stimulate cell division. Quick transporting systems and the release of S1P may be some of these mechanisms [54,59,98]. Notably, endothelial cells are quite resistant to malignant transformation as angiosarcoma is a rare type of cancer.

Not only S1P, but also SphK1 can be secreted by vascular endothelial cells [28]. Extracellular transport of SphK2 is unlikely, however SphK2 was recently found to have a role in the regulation of platelet aggregation, thrombosis, and cardio protection [68,109,195]. Secreted S1P favors not only egress and migration of immune cells, but also angio- and lymphangiogenesis in both normal and tumor microenvironment [22,23,126], which were also linked to increased lymph node metastasis in breast cancer patients [22,126].

Endothelial cell proliferation, directed migration, and/or chemotaxis are controlled by a sphingolipid network, specifically SphK/S1P and S1PRs which in turn can be activated by growth factors, cytokines, and hormones. For instance, VEGF and its tyrosine kinase receptors are essential for establishing the capillary bed within the developing intermediate villi during the first half of human pregnancy [196]. VEGF is the most potent mitogen known to stimulate angiogenesis [197]. Estrogen is also a very strong activator of angiogenesis and can stimulate expression of VEGF by the uterus during the normal reproductive cycle to facilitate angiogenesis in the placenta [196]. The endothelial effects of VEGF and estrogen were mediated by sphingolipids in endothelial cells [21,64]. During both VEGF and estrogen signaling, S1PR1 mediated the growth and migration of endothelial cells [21]. Indicating the leading role of this receptor, S1PR1-deficiency is lethal for mice, causing improper blood vessel formation and massive hemorrhaging [198].

S1P has a profound role in the regulation of immune functions [25]. The S1P signaling axis has been implicated in transduction of inflammatory signals [78,166,187]. Macrophages, dendritic cells, mast cells, T and B cells, and other lymphoid cell components demonstrate unique characteristics of the expression profile of S1PRs. The SphK/S1P pathway was implicated in the regulation of immune cell development and maturation, egress from lymphoid organs, recirculation and trafficking, tissue homing patterns and chemotactic responses during acute inflammatory responses, and general maintenance of homeostasis. Advances in the investigation of these processes have been recently reviewed and summarized [166,187]. For instance, S1P receptors modulate monocyte activity mediating CD40 and TNFα signaling against bacterial infection [199]. The sphingolipid network also mediated NF-κB activation and chemokine expression during recruitment of mononuclear cells into inflamed tissues [63,66,161,166]. However, it is unclear whether similar mechanisms can be triggered in the immune system during the elimination of transformed cancerous cells and the initiation of tumor growth. Maintenance of the S1P gradient between cells and blood plasma regulates lymphocyte trafficking leading to egress of cells from lymphoid organs and/or retention of specialized immune cells in inflamed tissue [78]. It remains to be identified whether the S1P gradient is being changed during spontaneous oncogenic transformation that also attracts specific immune cells (Figure 2). Creating a model of this process *in vivo* is complex and challenging. Considering that estrogen was shown to influence specific immune functions, further investigation is needed to test for a possible relationship between the SphK/S1P axis and estrogen-related regulation of immune responses related to inflammation and cancer initiation.

The role of estrogen in the regulation of normal plasma S1P levels was recently addressed. Liquid chromatography tandem mass spectrometry results showed that the plasma S1P levels were significantly higher in women than those in men and post-menopausal women within the age of 16–55 years old [69]. A similar analysis in C57 BL/6 mice confirmed the gender difference of plasma S1P levels. The study showed that estrogen markedly improves S1P synthesis by activating SphK1 and induces S1P export via activating ABCC1, G2, and Spns2 from the endothelium system, which may consequently lead to the gender difference of plasma S1P levels. S1P demonstrated prominent vasoactive actions in the maternal arteries of pregnant women [200]. Suggestively, estrogen may exert its vasculoprotective function via the SphK1/S1P/S1PR signaling axis, as pre-menopausal women are less affected by CVD than men or post-menopausal women [21,64,69].

Ordinarily high plasma S1P supports a function of the lipid in maintenance of low S1PR expression due to the constant binding of S1P to its receptors, followed by internalization and degradation of the complex. This suggestion, however, requires experimental confirmation. Taking into consideration the reported higher levels of S1PR1/S1PR3 in cancer cells compared to normal surrounding cells [35], it is tempting to suggest that high extracellular S1P and low levels of S1PR1/S1PR3 might represent an anti-cancer requirement. Supporting this hypothesis, decreased levels of circulating S1P were reported during prostate cancer progression [201]. This low S1P level was associated with decreased erythrocyte SphK1 levels that correlated well with high prostate specific antigen levels and lymph node status, and was suggested as an early marker for androgen independent tumors [201]. Suggestively, the level of S1P transporters must be higher in normal cells to provide quick release of S1P to interstitial fluids. Activities of S1P lyase and phosphatase might be increased to keep the level of S1P lowered in the cytoplasm of non-malignant cells. Accordingly, S1P lyase was predominantly expressed in the non-tumorigenic cell line MCF-12A [202]. Phosphatase activity would deliver a high level of sphingosine, which is a pro-apoptotic sphingolipid. The role of estrogen in the regulation of S1P phosphatase is unknown in normal and malignant cells, although the expression levels of S1P lyase and the presence of ethanolamine phosphate were significantly influenced by estrogens and phytoestrogen genistein in MCF-7 and MCF-12A cells [202].

Furthermore, SphK1 is often activated in very slow-dividing cells, including brain cells [203,204], confirming that generation of S1P in terminally differentiated cells is essential for the maintenance of not only structural and metabolic, but also signaling functions. SphK1 was shown to regulate the function of voltage-operated calcium channels in GH4C1 pituitary cells through the removal of pro-apoptotic sphingosine [203]. Overall, intracellular calcium fluxes were strongly associated with S1P signaling and SphK activation in different cells [5,130,136,157]. S1P facilitated glutamate secretion in hippocampal neurons, suggesting its potential involvement in the regulation of synaptic transmission [204]. The presence of significant Sphk1/2 abnormality and sphingolipid alterations was shown in the development of Parkinson’s [205] and Alzheimer diseases [166,206]. Notably, beneficial anti-apoptotic effects of estrogen were also mediated by SphK in brain cells. Experimental data indicated that glutamate-induced Tau hyperphosphorylation (glutamate toxicity) in SH-SY5Y cells was prevented by estrogen pre-treatment and required SphK activation [207].

**Figure 3:**
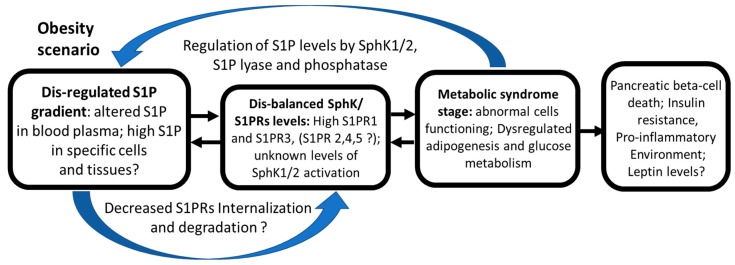
Obesity scenario: transformation and/or adaptation of sphingolipid signaling under condition of metabolic stress (hypothetical scheme). Sphingolipid signaling pathway was proposed to alter during metabolic stress in obese organisms. Blood plasma of obese patients was characterized by dis-regulated S1P gradient, decreased levels of S1P in blood plasma, but higher S1P in specific cells and tissues [102,107,209,210]. Higher S1PR1 and S1PR3 levels were also detected [68]. However, these findings require further confirmation. Black arrows indicate directions of S1P/S1PRs effects. Question marks indicate the gaps in our knowledge of SphK/S1P/S1PRs axis.

Sphingolipid-related research branched out in one more direction in the field of metabolic syndrome and obesity that are known to facilitate the development of cancer, diabetes, and various cardiovascular diseases. We have recently reviewed the role of sphingolipid signaling in the progression of insulin resistance and diabetes-associated pathologies [68]. Furthermore, obesity is an established risk factor for various types of adenocarcinomas, including breast cancer. Interestingly, obesity was marked by an increased S1P levels in humans and mice [68,107,208]. However, it was not established whether the increased plasma S1P is the cause or consequence of adiposity. Increased S1P levels in plasma could be an adaptation to metabolize and store lipids consumed during constant overeating. It could also be a natural response of an organism overloaded with adipose tissue and stored lipids, as an attempt to balance this overloading. Nevertheless, an obesity-related imbalance in sphingolipid metabolism, particularly an increased level of S1P in blood plasma, was suggested to impact the development of liver pathobiology and associated metabolic syndrome [208] (Figure 3). Increases in plasma levels of S1P might impact expression levels of S1PRs in many (possibly in all) blood circulating and endothelial cells, distort lymphocyte trafficking, and influence the direction of inflammatory responses. However, this suggestion requires future investigation.

One of the key cancer-related findings indicated that adipokine leptin stimulated expression of SphK1 in ER-negative breast tumors [107]. Furthermore, SphK1 mediated leptin-induced pro-inflammatory cytokine IL-6 secretion, linking obesity-associated inflammation to the sphingolipid network [209]. SphK1 deficiency and pharmacological inhibition increased markers of adipogenesis and adipose gene expression of the anti-inflammatory molecules IL-10 and adiponectin, as well as reducing adipose tissue macrophage recruitment and production of proinflammatory molecules TNFα and IL-6 [210]. Thus, SphK1 inhibition could be potentially beneficial in obese patients. However, the published data are controversial and marked by a high heterogeneity of sphingolipid effects [68,102,209,210] (Figure 3). Future clinical and *in vivo* studies should clarify the role of sphingolipids in the development and progression of obesity-linked pathologies.

## 11. Future Perspectives of Sphingolipid Anti-Cancer Strategy

It is important to conclude that SphK/S1P effects are not restricted to cancer promotion. On the contrary, a tightly balanced sphingolipid signaling network is essential for the maintenance of normal homeostasis in terminally differentiated cells and/or specialized cells, including pluripotent progenitors. SphK/S1P/S1PR network signaling is being transformed within tumor cells and their microenvironment (Figure 2). This conversion strongly resembles malignant adaptation, often called “cancer highjacked” transformation, of estrogen or growth factor signaling networks. For instance, numerous beneficial differentiation-related effects of estrogen were demonstrated in non-reproductive organs including bone, brain, gastrointestinal tissue [13,90,91,92,93], and the cardiovascular system [196]. However, malignant transformation of signaling turns estrogen into a pro-oncogenic growth factor for breast cancer cells.

Several decades of investigation of the estrogen signaling network resulted in the design of multiple specific estrogen receptor modulators (SERM) and antagonists. Some of these, including tamoxifen, are being widely used in cancer treatment, while others are being tested as potential anti-cancer drugs [91,92]. The tissue specific effects of estrogen are associated with exclusive triggering of ER isoforms, designated signaling pathways, and downstream mediators. Accordingly, specific ER modulators were developed to target tissue- and function-specific effects. Sphingolipid-related research also indicated the specificity of responses based on the type of SphK isoform and the combination of specific types of S1PRs expression. Thus, a detailed understanding of diversification of the sphingolipid cell signaling pathway, cell-specific sphingolipid proteomics, and its differentiation- or cancer-related function should also help to target cancer-cell exclusive biochemical reactions while avoiding harmful effects in normal tissues. Design and in vivo testing of specific SphK/S1PRs modulators along with ERs inhibitors should be addressed in future studies. 

The connection between the estrogen receptor modulators and sphingolipid network was recently revealed [211]. One of the promising cancer-preventive agents, phytoestrogen genistein, induced moderate intracellular ceramides accumulation, growth inhibition, and apoptosis in cultured B16 melanoma cells [212]. Genistein exerted inhibitory effects on the proliferation of estrogen-dependent MCF-7 cells. The agent was signaling as a stimulator of acid ceramidase ASAH1 transcription through a GPR30-related mechanism. The activation of this pathway promotes histone acetylation and the recruitment of phosphorylated ERα and specificity protein-1 to the ASAH1 promoter, ultimately resulting in increased ceramidase activity and the promotion of sphingolipid metabolism in breast cancer cells [213]. It is surprising that the influence of agents targeting ERs and SphK are rather mutual, as SphK inhibitor also affected ER signaling in human breast cancer cells [9,14,21,113]. The findings suggest that there is a potential protein-protein dual or tripartite interaction between ER and SphK that was overlooked and should be clarified in future studies.

## Abbreviations

EGFR	Epidermal growth factor receptors
SphK	Sphingosine kinase
S1P	Sphingosine-1-phosphate
S1PRn (*n* = 1–5)	Sphingosine-1-phosphate receptor
S1PR1	S1P receptor 1
S1PR3	S1P receptor 3
S1PR2	S1P receptor 2
S1PR4	S1P receptor 4
S1PR5	S1P receptor 5
IHC	Immunohistochemistry
MAPK	Mitogen-activated protein kinase
Erk1/2	Extracellular signal-regulated kinase 1/2
ER	Estrogen Receptor
Ca^2+^	Calcium ions
TAM	Tamoxifen
MPA	Medroxy-progesterone acetate
GPR	G-protein coupled receptor
hTERT	human telomerase reverse transcriptase
